# Características actuales y factores de riesgo de mortalidad en choque cardiogénico por infarto de miocardio en un hospital latinoamericano

**DOI:** 10.47487/apcyccv.v1i4.89

**Published:** 2020-12-31

**Authors:** Rosario Guzmán-Rodríguez, Gracia Polo-Lecca, Ofelia Aráoz-Tarco, Carlos Alayo-Lizana, Manuel Chacón-Diaz

**Affiliations:** 1 Servicio de Cardiología Clínica, Instituto Nacional Cardiovascular INCOR. Lima, Perú. Servicio de Cardiología Clínica Instituto Nacional Cardiovascular INCOR Lima Perú

**Keywords:** Choque Cardiogénico, Infarto del Miocardio, Mortalidad, Perú, Cardiogenic Shock, Myocardial Infarction, Mortality, Peru

## Abstract

**Objetivo.:**

Conocer las características clínicas y determinar los factores relacionados a mayor mortalidad en pacientes con choque cardiogénico (CC) por infarto de miocardio en un hospital de referencia peruano.

**Materiales y métodos.:**

Cohorte única prospectiva donde se evaluó la presentación, tratamiento y complicaciones de pacientes con CC por infarto de miocardio atendidos entre marzo 2019 a agosto 2020 en el Instituto Nacional Cardiovascular - INCOR. Se evaluaron los factores relacionados con mayor mortalidad hospitalaria y en el seguimiento, además del uso del *score* IABP *shock* II en la población de estudio.

**Resultados.:**

Cuarenta pacientes fueron incluidos, el 75% con CC por disfunción ventricular izquierda, la mayoría varones con edad de 75 (69-82) años. Un 50% de casos presentaron CC luego del ingreso a emergencia. Los pacientes estratificados mediante el score IABP *shock* II como bajo, intermedio y alto riesgo, tuvieron una mortalidad intrahospitalaria de 37,5; 71;4 y 91,6% respectivamente. La mortalidad intrahospitalaria fue 70%, mayor en mujeres, mayores de 75 años y en los que desarrollaron el CC durante la hospitalización. En el análisis univariado, el lactato sérico > 4 mmol/L al ingreso se relacionó con mayor mortalidad (HR:2,8; IC:1,6-3,6, p=0,009). La sobrevida hasta el término del estudio fue de 12,8%.

**Conclusiones.:**

EL CC por infarto de miocardio representa una entidad clínica de elevada mortalidad a pesar de la revascularización y el tratamiento disponible en nuestra realidad. El mayor predictor de mortalidad fue el valor de lactato sérico mayor a 4 mmol/L al ingreso. El *score* IABP *shock II* demostró ser un buen parámetro para estratificar el riesgo de muerte en nuestra población.

## Abstract (en)

**Objective.:**

To know the clinical characteristics and determine the related factors to higher in-hospital mortality in patients with cardiogenic shock (CS) due to myocardial infarction in a Peruvian reference hospital.

**Materials and methods:**

. We conducted a prospective single-center cohort study, to evaluate the clinical characteristics, treatment, and complications of patients with CS due to myocardial infarction from March 2019 to August 2020 at the Instituto Nacional Cardiovascular INCOR. Factors related to higher in-hospital mortality and during follow-up were evaluated. Also, the IABP shock II score was applied to stratify the cohort.

**Results.:**

Forty patients were included in the study, 75% of cases were due to left ventricular dysfunction, most of the men and with a median age of 75 (69-82) years. Fifty percent of cases presented CS after admission to the emergency room. Patients stratified by the IABP shock II score as low, intermediate, and high risk, had in-hospital mortality of 37.5%, 71.4%, and 91.6% respectively. In a hospital, mortality was 70%, higher in women, in those over 75 years old, and in those who developed CS during their hospitalization. Serum lactate > 4 mmol/L in univariate analysis was associated with higher mortality risk (HR: 2.8; IC:1.6-3.6, p=0.009). Survival to the end of the study was 12.8%.

**Conclusions.:**

CS due to myocardial infarction is a clinical entity with high mortality in spite of revascularization and the available treatment in our reality. The highest mortality predictor was the serum lactate at admission > 4 mmol/L. The IABP shock II score showed to be an accurate parameter to stratify the death risk in our population.

El choque cardiogénico (CC) es una entidad clínica caracterizada por la incapacidad del corazón de llevar la suficiente cantidad de sangre para suplir las demandas metabólicas en reposo a nivel tisular, requiere la presencia de un gasto cardiaco bajo y evidencia de hipoxia tisular en ausencia de hipovolemia ^1^. La definición del CC por infarto de miocardio requiere de todos los siguientes criterios: a) Hipotensión > 30 min; b) evidencia clínica de hipoperfusión tisular; c) evidencia clínica de elevación de las presiones de llenado de ventrículo izquierdo y, d) etiología cardiaca del choque ^1^.

A nivel mundial, la incidencia del CC en el caso del infarto de miocardio con elevación del segmento ST (IMCEST), se estima en un 5-10% y en caso del infarto de miocardio sin elevación del segmento ST (IMSEST) en un 2-4% ^2^. A pesar de los avances en la reperfusión temprana y en tratamientos para esta entidad, la mortalidad intrahospitalaria se ha mantenido en un rango de 55-60% ^1^. Existen parámetros clínicos y laboratoriales que pueden predecir el riesgo de muerte en los pacientes con CC desarrollados a partir del IABP-SHOCK II trial ^3^ e incluyen la edad > 75 años, historia de *stroke*, glucosa > 191 mg/dL, creatinina > 1,5 mg/dL, lactato > 5 mmol/L y estado de reperfusión (flujo TIMI), los que clasifican a los pacientes en bajo, moderado o alto riesgo de muerte.

En el Perú se ha reportado que el CC complica hasta el 10,9% de pacientes con IMCEST ^4^ con una mortalidad intrahospitalaria del 61% ^5^, pero aún no se han definido los factores de riesgo que están asociados al aumento de la mortalidad en nuestra población. Por lo que el objetivo del estudio es determinar las características relacionadas con mayor mortalidad intrahospitalaria y en el seguimiento en los pacientes con CC por infarto de miocardio en un centro de referencia nacional.

## Materiales y métodos

Cohorte única prospectiva en la que se evaluaron las características clínicas, epidemiológicas y laboratoriales de los pacientes admitidos al Instituto Nacional Cardiovascular INCOR, en Lima, Perú, con el diagnóstico de CC por síndrome coronario agudo y su relación con la mortalidad intrahospitalaria y en el seguimiento (mediana de once meses). El proyecto fue aprobado por el Comité de Ética Institucional.

La definición usada para catalogar a un caso como CC se hizo con la presencia de los siguientes criterios: 1) presión arterial sistólica (PAS) < 90 mmHg al menos por 30 min o la necesidad de uso de presores o inotrópicos para mantener la PAS > 90 mmHg; 2) signos clínicos de hipoperfusión o congestión pulmonar en ausencia de hipovolemia o arritmias.

Se incluyó a pacientes mayores de 18 años, con CC diagnosticado desde el ingreso al hospital o desarrollado durante la hospitalización y de etiología isquémica (IMCEST o IMSEST), se excluyeron a los pacientes con CC de causa valvular, obstructiva y por síndrome de Takotsubo. Todos los datos fueron obtenidos de manera prospectiva de los registros de la historia clínica de los pacientes y guardados en una base de datos electrónica específicamente creada para dicho fín. Las variables recolectadas fueron: sexo, edad, factores de riesgo cardiovascular, tipo de infarto, causa del CC (disfunción ventricular, complicación mecánica, parada cardiaca, tiempo de isquemia a reperfusión, tiempo desde el diagnóstico del CC hasta la reperfusión, localización de infarto, estrategias y éxito angiográfico de reperfusión, número de vasos con estenosis > 70%, número de vasos tratados y momento de tratamiento, terapia farmacológica, uso de soporte mecánico, uso de monitoreo hemodinámico invasivo, uso de ventilación mecánica, variables laboratoriales al ingreso y a las 24 h, muerte intrahospitalaria, estancia hospitalaria y sobrevida. Además, en el subgrupo de pacientes con IMCEST, a los que se realizó intervención coronaria percutánea (ICP) se les realizó la estratificación de riesgo según el *score* IABP SHOCK II ^6^ en bajo riesgo (puntaje 0-2); riesgo intermedio (puntaje 3-4), y alto riesgo (puntaje 5-9).

Las variables cualitativas se expresaron en frecuencias y porcentajes y en medias o medianas, y sus respectivas medidas de dispersión en caso de variables numéricas. La comparación entre ambos grupos se realizó mediante la prueba de chi cuadrado de Pearson (variables categóricas), T de Student (variables numéricas con distribución normal) y U de Mann Whitney (variables numéricas con distribución no normal). Se evaluaron los factores relacionados con mortalidad cardiovascular al año mediante análisis de regresión de Cox y se expresaron como HR y sus respectivos intervalos de confianza. El análisis estadístico se realizó utilizando el programa STATA 16. 

## Resultados 

**Figura 1:**
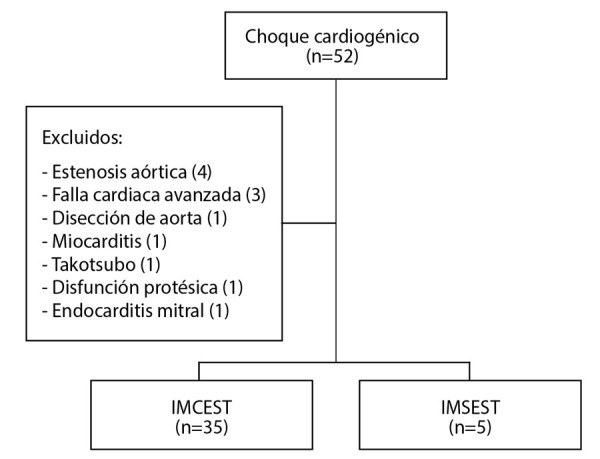
Flujograma de la población de estudio

Entre marzo 2019 y agosto 2020 se atendieron 52 pacientes con choque cardiogénico de diversa etiología, de ellos 40 (76,9%) fueron de causa isquémica (35 por IMCEST y 5 por IMSEST), los que conforman nuestra población de estudio **(**Figura 1**)**.

IMCEST: infarto de miocardio con elevación del segmento ST. IMSEST: infarto de miocardio sin elevación del segmento ST.

Las características generales y antecedentes de la población según tipo de infarto se presentan en la Tabla 1**.** Veinte pacientes (50%) llegaron a la emergencia en estado de CC y el resto lo desarrolló durante su estadía, en este último grupo el 30% presente CC posparada cardiaca, 25% por disfunción ventricular izquierda, 20% pos-PCI no exitosa, 15% por complicación mecánica, 5% poscoronariografía sin PCI en espera de cirugía y 5% por IMSEST.

**Tabla 1:** Características generales de la población de estudio según tipo de infarto

	Total (n=40)		IMCEST (n=35)		IMSEST (n=5)		p valor
	n	%	n	%	n	%	
Sexo masculino	31	77,5	28	80	3	60	0,311
Edad(mediana/RIQ)	75	69-82	75	70-83	82	68-82	0,851
HTA	25	62,5	21	60	4	80	0,633
DM2	14	35	11	31,4	3	60	0,322
EVC/TIA	7	17,5	7	20	0	0	0,563
ERC	7	17,5	6	17,1	1	20	1,000
Tabaquismo	8	20	8	22,8	0	0	0,563
Infarto previo	6	15	5	14,3	1	20	0,577
CRVM	1	2,5	0	0	1	20	0,125
ICP	3	7,5	3	8,6	0	0	1,000

IMCEST: infarto de miocardio con elevación del segmento ST. IMSEST: infarto de miocardio sin elevación del segmento ST. HTA: hipertensión arterial. DM2: diabetes mellitus tipo 2. EVC: enfermedad vascular cerebral. TIA: isquemia cerebral transitoria. ERC: enfermedad renal crónica. CRVM: cirugía de revascularización miocárdica. ICP: intervención coronaria percutánea. RIQ: rango intercuartil.

La etiología del CC en pacientes con IMCEST fue la disfunción ventricular izquierda (75%), síndrome posparada cardiaca (12,5%), complicación mecánica (7,5%) (ruptura de pared libre dos pacientes y de septum interventricular un paciente), y disfunción ventricular derecha (5%), mientras que en los pacientes con IMSEST fue la disfunción ventricular izquierda en tres casos (60%) y el síndrome posparada cardiaca en dos (40%).

El tiempo desde el inicio de síntomas isquémicos hasta la reperfusión en caso de IMCEST fue de doce horas (RIQ: 8,1 - 29,5), en el mismo grupo el tiempo puerta-balón fue de 80 min (RIQ:60-120 min). El tiempo desde el inicio del CC hasta la colocación de soporte mecánico (en los 22 casos que se colocó) fue de 100 min (RIQ: 25 - 660). El tiempo desde el diagnóstico de choque cardiogénico hasta el ingreso a nuestro hospital fue de 4,8 h (RIQ:2,1 - 13,1), el 17,5% de casos presentaron un tiempo en CC mayor o igual a doce horas al momento del ingreso.

### Reperfusión

En los 35 pacientes con IMCEST, el 54% fue de cara anterior y 46% de otras caras. Dieciocho pacientes (51,4%) fueron diagnosticados de CC luego del ingreso a emergencia. Seis pacientes (17,2%) recibieron fibrinólisis antes del ingreso y en 29 (83%) se realizó intervención coronaria percutánea (ICP), en cinco pacientes (14%) no se hizo ICP luego de la coronariografía: cuatro por necesidad de cirugía cardíaca (dos casos de enfermedad multivaso de indicación quirúrgica, un caso de ruptura de septum interventricular y otro caso por embolismo coronario por endocarditis), y uno por parada cardiorespiratoria (PCR) en sala de hemodinamia; en un paciente (3%) no se llegó a hacer coronariografía por PCR en sala de emergencia. 

De los 34 pacientes con coronariografía, el acceso más frecuente fue el radial (44%), luego el femoral (41%) y al final el braquial (8,8%). La arteria responsable del infarto (ARI) fue la descendente anterior en 53% de casos, coronaria derecha en 32,4% y la circunfleja en el 14,7%. La ARI en el 70,6% de casos estaba ocluida en el momento de la angiografía, y el flujo pos-ICP fue: TIMI 0 (10%), TIMI I (13.7%), TIMI II (24%) y TIMI III (51,7%). Se encontró que 22 pacientes (64,7%) presentaban enfermedad coronaria multiarterial y nueve de ellos (40%) fueron tratados con ICP de otro vaso además de la ARI. Dos casos (5,8%) tuvieron complicaciones del intervencionismo (taponamiento cardiaco y disección coronaria). Cinco pacientes (14%) requirieron de cirugía cardiaca (tres por complicación mecánica y dos por enfermedad multiarterial).

El 60% de los cinco pacientes con IMSEST llegaron con CC a la emergencia; se realizó coronariografía en el 100% de casos, encontrando, en todos, enfermedad coronaria multiarterial. El acceso predominante fue el femoral (80%), y se presentó una complicación por intervencionismo (disección de cayado aórtico). El 60% (tres casos) requirió de cirugía de revascularización, 20% ICP y en un caso no se realizó revascularización por PCR en sala de hemodinámica.

### Soporte farmacológico y no farmacológico

Al 80% de los pacientes del estudio se les colocó balón de contrapulsación intraaórtico (BIAo), las características de este y otras terapias de soporte se presentan en la Tabla 2**.** El uso de BIAo se complicó en un caso con isquemia del miembro inferior. El único caso que requirió de asistencia mecánica ventricular de corta duración (ECMO) fue por IMCEST de cara anterior luego de colocación de BIAo. En cuanto a la medicación administrada durante las primeras 24 h de la hospitalización se encontró el uso de noradrenalina en el 100% de los casos, con una mediana de dosis de 0,3 ug/k/min (RIQ: 0,12 a 0,9 ug/k/min); dobutamina en 85% de casos con una dosis promedio de 5,9 ± 2,3 ug/k/min; dopamina en 20% con dosis promedio de 8,5 ± 3,1 ug/k/min.

**Tabla 2:** Soporte administrado a los pacientes en CC según tipo de infarto

	Total (n=40)		IMCEST (n=35)		IMSEST (n=5)	
	n	%	n	%	n	%
BIAo precoronariografía	7	17,5	4	11,4	3	60
BIAo poscoronariografía	25	62,5	23	65,7	2	40
No BIAo	8	20	8	22,8	0	0
Ventilación mecánica	39	97,5	34	97,1	5	100
Catéter de AP	26	60	24	68,5	2	40
ECMO	1	2,5	1	2,9	0	0

IMCEST: infarto de miocardio con elevación del segmento ST. IMSEST: infarto de miocardio sin elevación del segmento ST. BIAO: balón de contrapulsación intraaórtico. AP: arteria pulmonar. ECMO: oxigenación por membrana extracorpórea.

### Laboratorio

Los valores de lactato sérico del ingreso (mediana: 2,9 mmol/L, RIQ: 1,9-6,2) disminuyeron en un 48% a las 24 h de tratamiento (mediana: 1,5 mmol/L, RIQ: 1,1-3,1); los valores de creatinina al ingreso (mediana: 1,6 mg/dL, RIQ=0,9 -2,1) se incrementaron a las 24 h en un 37,5% (mediana: 2,2 mg/dL, RIQ= 0,9-3,4); lo mismo en el caso de las transaminasas que subieron en 9%; en el caso de la hemoglobina hubo una disminución de un 18% (promedio 11±3 a 9 ±2 mg/dL) a las 24 h.

### Estratificación de riesgo y mortalidad

**Figura 2:**
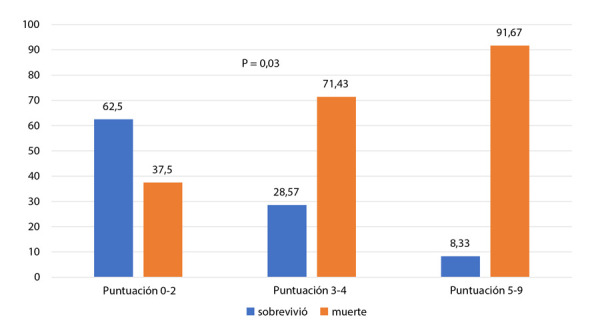
Mortalidad según estratificación de riesgo con score IABP shock II.

Se realizó la estratificación del riesgo con el cálculo del score IABP *shock* II en los pacientes con IMCEST a los que se realizó ICP (29 pacientes), encontrando un 29,6% con bajo riesgo, 25,9% con riesgo intermedio y 44,4% con alto riesgo. La mortalidad intrahospitalaria (MIH) fue mayor en pacientes estratificados como alto riesgo según dicho *score* (p=0,034) **(**Figura 2**).**

**Figura 3:**
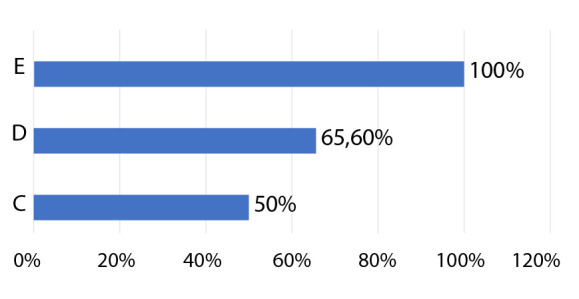
Porcentaje de mortalidad intrahospitalaria según clasificación SCAI máxima alcanzada.

Tomando en cuenta la clasificación de la *Society for Cardiovascular Angiography and Interventions* (SCAI) ^7^, encontramos que inicialmente el 30%, 55% y 15% de casos estaban en estadio C, D y E respectivamente, lo que cambio a 5%, 80% y 15% de los mismos estadios durante la hospitalización (el aumento de casos en estadio D se caracterizó por el aumento del soporte farmacológico por falta de respuesta inicial). La mortalidad intrahospitalaria fue mayor en pacientes en estadio E comparándolo a estadios C y D (p=0,223) **(**Figura 3**).** El resumen de las características, tratamientos y mortalidad se presentan en la Figura Central**.**

**Figura central:**
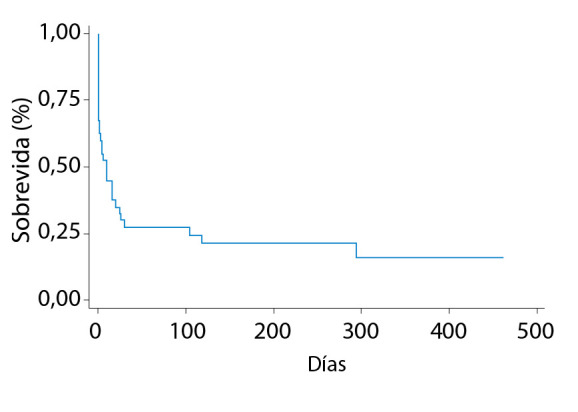
Características clínicas, terapéuticas, clasificación y mortalidad de pacientes con choque cardiogénico por infarto de miocardio.

La MIH fue del 70% (71,4% en IMCEST y 60% en IMSEST, p=0,627), mayor en mujeres que en varones (77% versus 67%). La MIH en pacientes mayores de 75 años fue 71,4% (68,4% en menores). Las mujeres mayores de 75 años tuvieron una mortalidad de 77% y los varones mayores de 75 años de 66% (HR:1,16, IC: 0,7 - 1,1, p=0,44). Un 75% de pacientes que desarrollaron CC durante la hospitalización fallecieron en comparación con 65% con CC desde el ingreso a emergencia (p=0,490). Los pacientes con CC de más de 12 h de evolución tuvieron una mortalidad de 71,5%, mientras que fue 68,9% en los CC de menos de 12 h de evolución (p=0,641).

**Figura 4:**
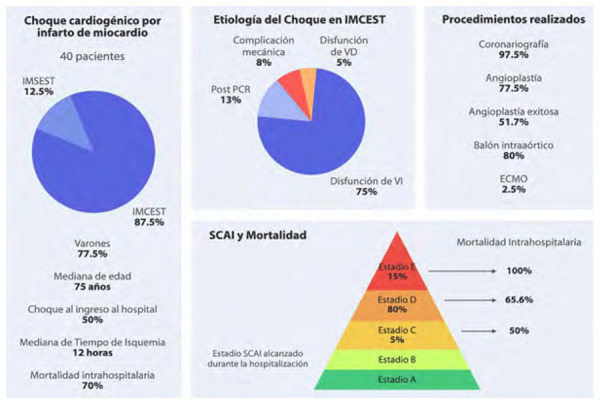
Curva de Kaplan Meier con la sobrevida de los pacientes atendidos por CC.

En el seguimiento, la mortalidad por causa cardiovascular a 30 días permaneció similar (70%), pero en el seguimiento hasta los 16 meses se incrementó a 79,5% (82,3% en IMCEST y 60% en IMSEST, p=0,268). La sobrevida en el seguimiento hasta una media de 11 meses fue de 12,8 % (11,8 en IMCEST y 20% en IMSEST, p=0,517) **(**Figura 4**).**

El análisis de regresión de Cox de algunos factores de riesgo mostró tendencia a mayor mortalidad en el seguimiento en mujeres, mayores de 75 años, flujo TIMI pos-ICP < 3, CC intrahospitalario y lactato mayor a 4 mmol/L, solo este último con significancia estadística en análisis univariado. Sin embargo, al realizar el análisis multivariado ninguno de los anteriores demostró ser una variable predictiva independiente **(**Tabla 3**).**

**Tabla 3:** Análisis de regresión de Cox de las características clínico-laboratoriales asociadas con mortalidad por choque cardiogénico en el seguimiento al año

	Análisis univariado		
	HR	IC 95%	p valor
Femenino	1,6	0,2 - 9,5	0,554
TIMI < 3	1,1	0,5 - 2,5	0,815
Lactato > 4 (ingreso)	2,87	1,3 - 6,3	0,009
Edad > 75 años	1,15	0,2 - 4,4	0,836
Uso de BIAo	1,44	0,5 - 3,7	0,437
CC durante hospitalización	1,61	0,8 - 3,4	0,147

BIAo: balón de contrapulsación intraaórtico. CC: choque cardiogénico. HR: hazard ratio. IC: intervalo de confianza

La estancia hospitalaria fue de 10,5 días (RIQ: 1,5 - 22) y la estancia en unidad de cuidado intensivo fue de 7,5 días (RIQ: 1 - 17,5). En seis pacientes (15%) se presentaron complicaciones infecciosas que llevaron a choque séptico y cinco (12,5%) requirieron de terapia de reemplazo renal (hemodiálisis).

## Discusión

En este estudio se encontró que la mortalidad intrahospitalaria sigue siendo elevada (70%) y es mayor a la encontrada hace cuatro años en el registro PERSTEMI ^5^^)^ (61%). Entre los factores relacionados con mayor mortalidad, solo el lactato > 4 mmol/L al ingreso fue significativo (en el análisis univariado), aunque la edad > 75 años, el sexo femenino, la reperfusión inadecuada (flujo TIMI posintervención < 3) y la aparición del CC durante la hospitalización se presentaron como variables probablemente asociadas con mayor mortalidad, pero sin significancia estadística.

Tal como se menciona en registros internacionales, el IMCEST es más frecuente que el IMSEST como causa de CC, y es más frecuente en edad avanzada y en el sexo masculino ^6^^,^^8^. En algunos estudios la edad es considerada como un factor de riesgo independiente; así, los pacientes mayores de 75 años tienen mayor mortalidad intrahospitalaria y en el seguimiento a largo plazo, además, es una variable considerada en varios *scores* de riesgo ^3^^,^^6^^,^^9^, lo que ha sido verificado al aplicar el *score* IABP *Shock* II en nuestro estudio.

La experiencia latinoamericana muestra que alrededor de la mitad de los pacientes con SC llega a la emergencia en estados Killip Kimbal I a III (44% en el registro argentino) ^8^, el mismo estudio encontró que uno de cada cuatro casos desarrolla el CC luego de las 24 h de iniciado el infarto. En nuestro estudio encontramos datos similares en cuanto al porcentaje de casos que llegan a emergencia sin estado de CC (50%), siendo las razones por las que presentaron CC posteriormente la arritmia ventricular y parada cardiaca, y la falta de reperfusión adecuada (evaluada por un flujo TIMI post ICP < 3). En otros estudios el 59% de pacientes presentaron CC temprano (< 48 h después del infarto de miocardio) ^10^^,^^11^ encontrando además que el CC intermedio o tardío (después de 48 h) tenía mayor mortalidad a los 30 días (80% vs. 45%, p <0,05) ^11^.

Las guías de práctica clínica recomiendan la reperfusión precoz en los pacientes con CC posinfarto,^12^^,^^13^; sin embargo, nuestros tiempos aún no son óptimos, el tiempo desde el inicio de síntomas isquémicos hasta la reperfusión, en caso de IMCEST, fue de 12 h, mayor a las seis horas promedio descritas en el registro PERSTEMI ^4^, el mayor tiempo de isquemia ya es un factor que empeora la evolución del paciente y puede predisponer a la aparición del CC. Nuestros datos podrían reflejar las demoras tanto en el diagnóstico como el manejo de los pacientes con IMCEST y debe orientarnos a mejorar el tratamiento, prevención e identificación de los pacientes en riesgo de CC.

Thiele *et al.* ^(^^14^ reportaron aproximadamente 70 a 80% de pacientes en CC con enfermedad coronaria multivaso, similar a lo hallado en este estudio (64,7%) y a otros registros latinoamericanos ^8^^,^^15^. Si bien es cierto que desde los resultados del CULPRIT SHOCK ^16^^)^ se prefiere una estrategia de tratar solo la arteria culpable y, posiblemente, una revascularización por etapas, ya que se mostró beneficio en reducción de mortalidad a los 30 días y terapia de reemplazo renal en este grupo de pacientes con enfermedad multiarterial en CC, encontramos que hasta en 40% de casos de nuestra cohorte se realizó revascularización percutánea de las arterias no relacionadas al infarto en el mismo procedimiento, sin que esto mejore la mortalidad intrahospitalaria.

Actualmente, en el escenario de CC, es más frecuente la revascularización percutánea que la quirúrgica, en el ensayo clínico IABP SHOCK II ^1^^,^^6^ la cifra de cirugía fue de un 4%, en el nuestro fue 10,4% lo que refleja el tratamiento de los pacientes con complicación mecánica del infarto y casos de pacientes con enfermedad multiarterial con ARI no reperfundida (TIMI < 3). Es importante mencionar que a pesar de que más del 80% de pacientes accedieron a revascularización percutánea, el éxito de esta (flujo TIMI final 3) se alcanzó solo en un poco más de la mitad de los casos. Estudios previos demuestran que el flujo TIMI inadecuado después de la angioplastia es un predictor independiente de mortalidad ^9^^,^^17^, lo que puede explicar la mortalidad elevada en nuestra cohorte.

Dada la fisiopatología del CC, una estrategia de manejo es la descarga ventricular con el uso de soporte circulatorio mecánico (SCM), que permite la reducción del consumo de oxígeno del miocardio, y mejora del gasto cardiaco, lo que aumentaría la perfusión coronaria y sistémica ^1^^,^^18^. En ese sentido, el uso del BIAo llegó casi al 80% de casos, que difiere bastante de lo encontrado en Argentina (37%) ^8^ o en Chile (16,5%) ^15^ solo por mencionar registros vecinos, a pesar de que el estudio IABP *shock* II no demostró el beneficio de su uso ^6^. De este grupo de usuarios de BIAo casi 22% fueron luego de una arritmia ventricular o por complicación mecánica del infarto. La mayoría fueron colocados luego de la ICP y no mejoraron el pronóstico del paciente. De igual manera, el uso de otros dispositivos como ECMO representó un pequeño porcentaje (2,5%) similar a lo reportado en Argentina (2,4%) ^8^. Aún no existe un tiempo óptimo de colocación del SCM, se asume que reduciendo el tiempo a su aplicación se podría optimizar el estado del paciente antes del desarrollo de choque irreversible ^19^. En el DanGer registry ^18^ la colocación del dispositivo se realizó inmediatamente después de la primera confirmación del CC, por lo que, si el paciente estuvo en choque antes de la ICP, recomiendan colocar el dispositivo antes de esta, considerando que utilizaron un dispositivo de soporte (Impella®) con el que no contamos en nuestra realidad.

La elevación del lactato sérico refleja hipoperfusión sistémica y disfunción orgánica, el punto de corte óptimo y en el tiempo ideal para su medición aún no está definido. En algunos estudios se le considera como factor pronóstico de mortalidad ^20^. Furneau *et al*. ^(^^21^ encontraron que un valor de corte de 3,1 mmol/L para el lactato después de 8 h mostró la mejor discriminación para evaluar el pronóstico temprano en el CC comparándolo con el valor de lactato basal y la depuración de lactato. Para nosotros, el valor que se correlacionó con mayor mortalidad fue el lactato > 4 mmol/L al ingreso, lo que puede traducir el impacto de la falla circulatoria a nivel periférico y, por ende, la probabilidad de mayor mortalidad.

A pesar de la revascularización temprana y otras estrategias de tratamiento, el CC continúa teniendo elevada mortalidad; la mortalidad intrahospitalaria, y a los 30 días,, en nuestro estudio fue mayor a la del registro IABP *shock* II ^6^ (40%) y a lo reportado en Argentina (54%) ^8^ o Chile (40,8%) ^15^ y similar a la realidad de México, donde la mortalidad global fue de un 80% ^17^. Las causas de la mayor mortalidad en nuestra cohorte pueden estar relacionadas a que casi la mitad de los pacientes con IMCEST no tuvieron reperfusión exitosa (TIMI < 3), casi el 20% tuvieron más de 12 h de CC al momento del manejo en el hospital o la demora en promedio > 12 h en la reperfusión del IMCEST (generalmente relacionada con demoras en el traslado de los pacientes); además que el 95% de la población de estudio presentó en algún momento de su hospitalización características de CC «en deterioro» y «extremo» (estadio D y E respectivamente de la clasificación SCAI) los que están relacionados con mayor mortalidad intrahospitalaria ^22^, por lo reciente de esta clasificación desconocemos el porcentaje de este grupo de pacientes de alto riesgo en los estudios argentino y chileno ^8^^,^^15^^)^ para su comparación.

El uso del *score* IABP *shock* II resultó de utilidad en predecir mayor mortalidad a mayor puntaje del *score*, pero no podemos validar su eficacia debido al tipo de estudio, en todo caso es un arma más para poder decidir estrategias de manejo más agresivas y precoces en pacientes de alto riesgo. Algunos estudios encuentran por análisis multivariado que la edad avanzada, hipotensión, coma profundo, insuficiencia cardíaca y lesión de tronco de coronaria izquierda son factores independientes de mortalidad a 30 días ^23^. En Chile encontraron como predictores de mortalidad intrahospitalaria y global a la fracción de eyección del ventrículo izquierdo < 30% y la presencia de 2 o más vasos enfermos ^15^. A pesar de encontrar una tendencia a mayor mortalidad, no encontramos relación significativa entre la edad, sexo, flujo TIMI posintervención, ni el uso de BIAo con la mortalidad, lo que podría deberse a nuestro tamaño muestral reducido.

El estudio presenta varias limitaciones, la primera es que se trata de un estudio unicéntrico de un hospital de referencia a nivel nacional, por lo que no refleja las características ni el manejo del CC en el Perú; el reducido número de casos no permite hacer inferencias estadísticas adecuadas, como regresión logística multivariada para conocer los factores relacionados independientemente a mayor mortalidad; no contamos con datos hemodinámicos adecuados en la población, ya que a pesar del uso de catéter de Swan Ganz muchos datos no fueron consignados en la historia clínica (índice cardiaco, poder cardiaco, índice de pulsatilidad de arteria pulmonar, etc.) para ser evaluados como factores relacionados con mortalidad. Solo encontramos un caso de CC por infarto de miocardio con asistencia con ECMO, por lo que el estudio no nos permite evaluar si el uso de este dispositivo puede estar relacionado con mayor o menor mortalidad. Algunas variables de importancia como las complicaciones de sangrado no fueron tomadas en cuenta por falta de datos en los registros médicos.

## Conclusiones

El CC por infarto de miocardio representa una entidad clínica de elevada mortalidad intrahospitalaria y en el seguimiento, a pesar de la revascularización y el soporte de tratamiento disponible en nuestra realidad. El lactato sérico en el análisis univariado fue un factor relacionado con mayor mortalidad intrahospitalaria. El score IABP II estratificó adecuadamente el riesgo de muerte de la población de estudio

Se debe plantear estrategias para optimizar el tratamiento de los pacientes con infarto de miocardio, desde la reperfusión temprana, el reconocimiento precoz del estado de pre choque cardiogénico ( SCAI- B) y el diagnóstico y estratificación de riesgo del paciente con CC, para que se pueda dar prioridad en el traslado hacia centros con capacidad resolutiva en el manejo de esta entidad, uno de los primeros pasos deberia ser la creación de una red de infarto a nivel nacional.
